# The major allergen Der p 2 is a cholesterol binding protein

**DOI:** 10.1038/s41598-018-38313-9

**Published:** 2019-02-07

**Authors:** Kavita Reginald, Fook Tim Chew

**Affiliations:** 1 Department of Biological Sciences, Sunway University, Bandar Sunway, 47500 Selangor, Malaysia; 2 Allergy and Molecular Immunology Laboratory, Functional Genomics Laboratories, Department of Biological Science, National University of Singapore, Block S2, Level 5, Science Drive 4, Singapore, 117543 Singapore

## Abstract

Der p 2 is a major dust mite allergen and >80% of mite allergic individuals have specific IgE to this allergen. Although it is well characterized in terms of allergenicity, there is still some ambiguity in terms of its biological function. Three-dimensional structural analysis of Der p 2 and its close homologues indicate the presence of a hydrophobic cavity which can potentially bind to lipid molecules. In this study, we aimed to identify the potential ligand of Der p 2. Using a liposome pulldown assay, we show that recombinant Der p 2 binds to liposomes prepared with exogenous cholesterol in a dose dependent fashion. Next, an ELISA based assay using immobilized lipids was used to study binding specificities of other lipid molecules. Cholesterol was the preferred ligand of Der p 2 among 11 different lipids tested. Two homologues of Der p 2, Der f 2 and Der f 22 also bound to cholesterol. Further, using liquid chromatography-mass spectrometry (LC-MS), we confirmed that cholesterol is the natural ligand of Der p 2. Three amino acid residues of Der p 2, V104, V106 and V110 are possible cholesterol binding sites, as alanine mutations of these residues showed a significant decrease in binding (p < 0.05) compared to wild-type Der p 2. These results provide the first direct experimental evidence that Der p 2 binds to cholesterol.

## Introduction

Group 2 allergens from the *Dermatophagoides* house dust mites causes IgE-mediated responses in over 80% of the dust mite allergic individuals^1,2^ and are therefore classified as major allergens. For the last 20 years, researchers have been interested to uncover the biological role of group 2 dust mite allergens. Initial reports on the binding of Der p 2 to the surface of *E. coli*^3^ and the presence of Gram-negative bacterial DNA in washed *D. pteronyssinus* mites^4^ led to the hypothesis that Der p 2 may be involved in the mite’s innate antibacterial defence system. Later, studies on the identification of the ligand of group 2 allergens were focused on lipopolysaccharide (LPS), which is a major component of bacterial cell wall. LPS also was shown to bind to MD-2, a protein which shared moderate sequence similarity (11% identity, 29% similarity) to Der p 2, and belonged to the same ML (MD-2 related lipid binding) domain family as group 2 allergens. Based on the high sequence similarities between Der p 2 and Der f 2 (88% identity), it would be expected that both proteins would behave in a similar manner in terms of ligand binding. Surprisingly, data from the LPS-binding experiments showed that Der p 2 bound weakly to LPS^5^, whereas Der f 2 bound to LPS at nanomolar affinities^6^.

Among the various proteins that belong to the ML domain family, Der p 2 shows the highest sequence similarity to NPC2^7^ (23.5% identity, 44% similarity). The structures of group 2 allergens and NPC2 are made up of a single domain β-sandwich protein, with 6 anti-parallel β-strands stabilized by 3 disulfide bonds^8–10^. The crystal structure of Der p 2^8^ shows the presence of two distinct elongated fragments of high electron density within its hydrophobic cavity, which, in their dimensions, could correspond to aliphatic chains of 14–16 carbon atoms. Since the 3D structures of Der p 2 and NPC2^10^ show high similarity, and NPC2 has been reported to bind cholesterol at nanomolar affinities^11^, we hypothesized that the ligand of Der p 2 could likely to be a lipid with close molecular similarity to sterols. Using well established lipid binding assays and mass spectrometry, we show direct evidence that Der p 2 is a cholesterol binding protein. In addition, we also show evidence that homologues of Der p 2, specifically Der f 2 and Der f 22, a paralogue of Der f 2^12^, binds to cholesterol.

## Results

### Der p 2 binds to liposomes with exogenous cholesterol in a dose-dependent fashion

**Figure 1:**
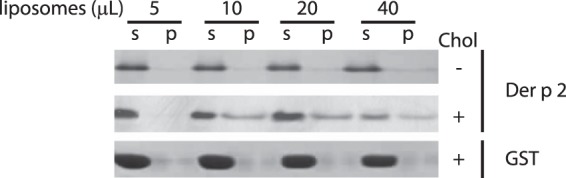
Liposome pull down assay. Liposomes with a fixed diameter of 0.2 μm were prepared using bovine brain lipid extract in HEPES-KCl buffer with 0.3 M sucrose, with (+) or without (−) exogenous cholesterol (20% w/w). Increasing amounts of liposomes (5, 10, 20 or 40 μL of a 2 mg/mL lipid suspension) were incubated with 50 μg of Der p 2, or glutathione-S-transferase (GST) for 30 minutes at 37 °C. The incubation mixture was then centrifuged at 30000 rpm, and the resulting supernatant (s) and pellet (p) were separated on a 12% SDS-PAGE gel. The gel was then stained with Coomasie Blue to visualize the amount of proteins bound to the liposome (p), or unbound (s). Full-length gels are presented in Supplementary Fig. S1.

A liposome binding experiment was carried out to investigate the binding of recombinant Derp-2 (rDer p 2) to unilamellar lipid vesicles. Crude bovine brain lipid extract, which contains approximately 10% phosphatidylinositol, 50% phosphatidylserine, and several other brain lipids was used as a lipid source. Liposomes with a defined size (0.2 μm in diameter) were prepared in HEPES-KCl buffer and incubated with rDer p 2. Bound protein was separated from free protein by centrifugation and separated by SDS-PAGE. It was observed that rDer p 2 weakly bound to liposomes in a dose dependent fashion (Fig. 1, top panel, Supplementary Fig. S1). Binding to liposomes was substantially enhanced when exogenous cholesterol (20% w/w) was included in the liposomal membrane (Fig. 1, middle panel). This indicated that cholesterol may be a ligand of rDerp2. Control experiments using glutathione-S-transferase (GST) in the same assay showed no significant protein binding to the liposomes with exogenous cholesterol (Fig. 1, bottom panel).

### Binding of rDer p 2 to purified lipids

**Figure 2:**
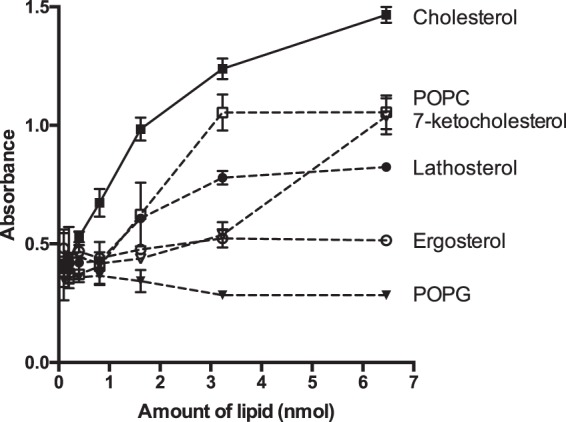
Binding of recombinant Der p 2 to six lipid molecules that include sterols and phospholipids. Lipids were coated (in serial dilutions) onto microtiter plates and incubated with 0.5 μg/mL of Der p 2. The bound Der p 2 was then probed with anti-Der p 2 IgG antibodies, followed by anti-IgG linked to alkaline phosphatase. Absorbance was measured at 405 nm after adding the PnPP substrate. Standard errors of duplicate experiments are shown. POPC, palmitoyloleoylphosphatidylcholine; POPG, palmitoyloleoylphosphatidylglycerol.

An ELISA based assay based on Kobayashi *et al*.^13^ was next used to characterize the specificity of Der p 2-lipid interactions. Various lipids were immobilized and assayed with recombinant Der p 2 followed by detection by polyclonal anti-Der p 2 antibodies. The panel of lipids consisted of sterols, phospholipids, triacylglycerols and sphingiolipids, thus representing a wide variety of different lipid chemistries^14^. Der p 2 exhibited binding to cholesterol consistent with the results from the liposome sedimentation assay (Fig. 1). From the eleven closely related sterols tested, only lathosterol and 7-ketocholesterol displayed a moderate degree of binding to rDer p 2 (Fig. 2). The other nine sterols, ergosterol, cholesteryl oleate, β-sitosterol, 5β, 6β-epoxycholestan-3β-ol, 24-hydroxycholesterol, campesterol, stigmasterol, 7α-hydroxycholesterol and coprostanol did not bind to rDer p 2 (Fig. 2, Supplementary Fig. S2A,B).

Five non-sterol lipids, palmitoyloleoylphosphatidylcholine (POPC), palmitoyloleoylphosphatidylglycerol (POPG), ceramide ((2 S,3 R,4E)-2-acylamino-1,3-octadec-4-enediol), triacylglycerol (TAG; 1,2-dioleoyl-3-palmitoyl-sn-glycerol) and palmitoyloleoylphosphatidylethanolamine (POPE) were also tested for their ability to bind to Der p 2. Of these, only POPC showed binding to Der p 2, albeit to a lower extent when compared to cholesterol (Fig. 2, Supplementary Fig. S2A).

### Cholesterol is the natural ligand of Der p 2

**Figure 3:**
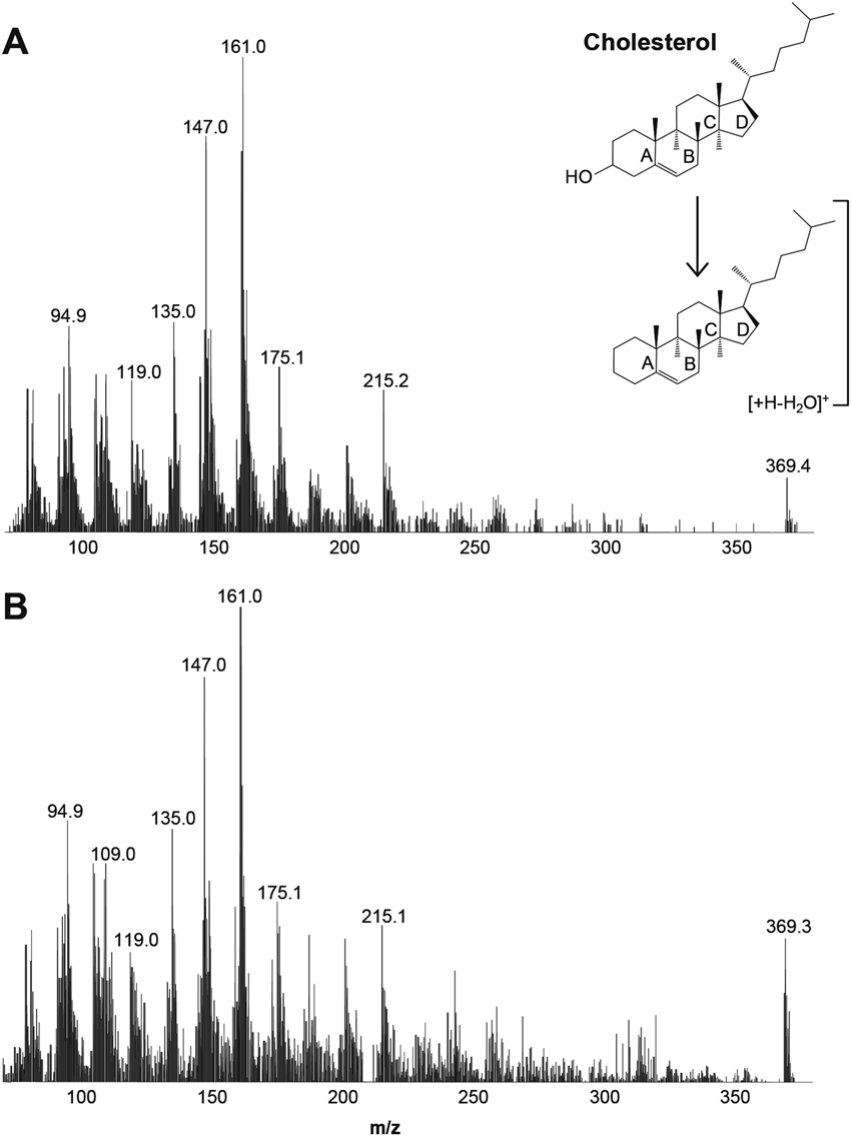
LC-MSMS analysis of cholesterol. (**A**) Cholesterol standard was separated on a reversed phase column followed by atmospheric pressure chemical ionization (APCI) mass spectrometry on a triple quadrupole instrument. Ions at m/z 369 correspond to dehydrated singly charged cholesterol in positive mode. The parent ion was selected in the first quadrupole and fragmentation induced with a collision energy of 40ev. This leads to product ions which originate from various parts of the sterol molecule, including the C and D ring. (**B**) Tandem mass spectrum using the same experimental conditions and an organic extract derived from recombinant Der p 2 (rDer p 2).

Next, liquid chromatography (LC) and mass spectrometry (MS) were used to determine the identity of the natural lipid ligands bound to Der p 2. In positive mode APCI, a prominent ion at m/z 369 is observed at 3.5 min when synthetic cholesterol standard is introduced into the LC-MS system. Cholesterol ions, [M + H]+, m/z at 387, lose one water molecule, thus m/z 369 [M + H-H2O]+ is the predominant parent ion for cholesterol under these conditions. Figure 3A shows tandem mass spectrometry (MSMS) and collision induced dissociation of cholesterol standard. Fragmentation of the parent ion at m/z 369 mainly occurred at C-ring and produced two major daughter ions at m/z 161.0 and 147.0, and m/z 175.1, respectively. Ions at m/z 215.2 originate from cleavages in the D-ring. To identify the natural lipid ligand of rDer p 2, its lipid fraction was extracted using chloroform/methanol (1:1) organic solvents, and analysed under the same parameters as synthetic cholesterol standards using LC-MS. The organic extract of rDer p 2 contained ions at m/z 369 that (i) have the same elution time as cholesterol standard, and (ii) show the same fragmentation pattern as cholesterol standard (Fig. 3B), indicating the presence of cholesterol in extracts of rDer p 2. Comparable results were observed when the lipid extract of nDer p 2 were used in place of rDer p 2 (data not shown), demonstrating that cholesterol is the natural lipid ligand of Der p 2. The presence of other potential lipids in the organic extract was also investigated using electrospray ionization which is generally used for polar compounds. Only low amounts of common glycerophospholipid were detected among a number of solvent peaks. Quantification (using deuterated internal standard) indicated that the cholesterol content was >10 times higher than that of other polar lipids such as phosphatidylcholine (POPC) (data not shown).

### Native Der p 2 and homologues of Der p 2 binds to cholesterol

**Figure 4:**
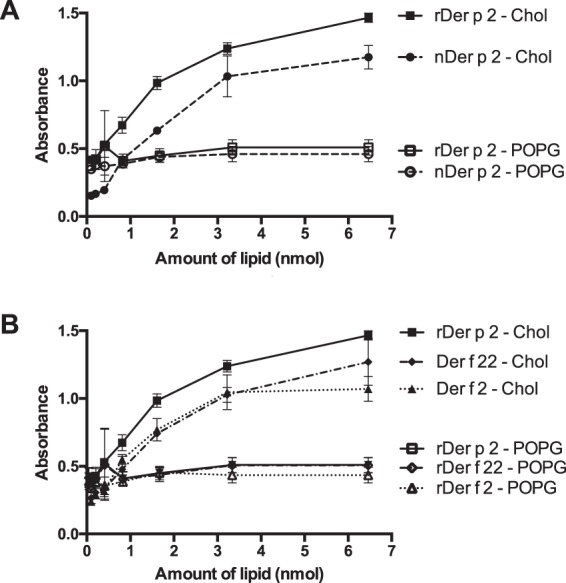
Binding of (**A**) native Der p 2 (nDer p 2) and recombinant Der p 2 (rDer p 2) and (**B**) rDer p 2, rDer f 2 and rDer f 22 to cholesterol or POPG (palmitoyloleoylphosphatidylglycerol). Cholesterol or POPG was coated on microtiter plates in serial dilutions and incubated with 0.5 μg/ mL of native or recombinant allergens. The bound proteins were then probed with corresponding rabbit polyclonal IgG antibodies (anti-Der p 2, anti-Der f 2 or anti-Der f 22), followed by anti-IgG linked to alkaline phosphatase. Intensity of the colorimetric reaction was measured at 405 nm, after the addition of the PnPP substrate. Mean and standard errors of duplicate experiments are shown.

We next assayed the binding of the recombinant and native forms of Der p 2 to cholesterol to evaluate any differences in dose-dependent binding. Both forms of Der p 2 bound to cholesterol in a dose-dependent manner, with nDer p 2 binding at lower cholesterol amounts compared to the recombinant form (Fig. 4A). Two allergens, Der f 2 and Der f 22 which are structural homologues of Der p 2, also showed dose-dependent binding to cholesterol, but not POPG in the same ELISA-based lipid assay (Fig. 4B).

### Identification of potential cholesterol binding sites in Der p 2 via site directed mutagenesis

**Figure 5:**
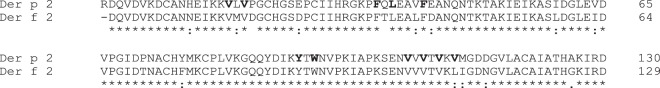
Multiple alignments of the amino acid sequences of Der p 2 and Der f 2 coded by their mature proteins. Sequences of Der p 2 and Der f 2 show 88% amino acid identity. Residues lining the cavity of Der p 2 which were chosen for generation of alanine mutants are bold faced. Gaps (represented by dashes) were included for optimal alignment using Clustal O (v1.2.4). An asterisk (*) indicated the positions with fully conserved residues, a colon (:) indicated conservation between groups of strongly similar properties, and a period (.) indicated conservation be-tween groups of weakly similar properties.

**Figure 6:**
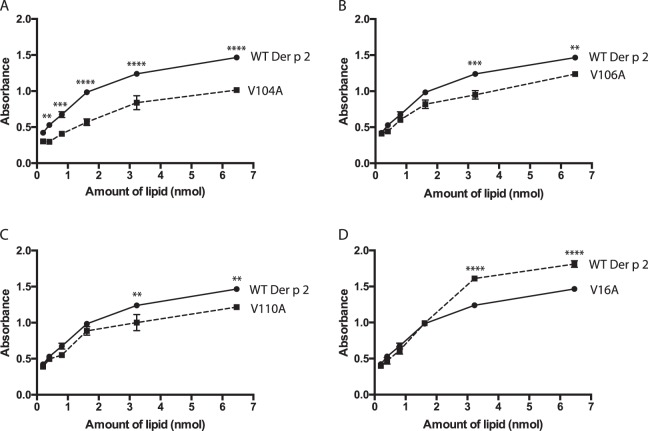
Binding of single site directed mutants of Der p 2 to cholesterol. Binding of mutants (**A**) V104A, (**B**) V106A, (**C**) V110A resulted in significant reduction in cholesterol binding as compared to wild-type Der p 2, however mutant (**D**) V16A indicates an increase in cholesterol binding. The binding of mutants to cholesterol was evaluated using the lipid ELISA assay. Mean and standard errors of duplicate experiments is shown.

Eleven amino acid residues lining the hydrophobic cavity of Der p 2 were individually mutated to alanine in order to assess their contributions to cholesterol binding of Der p 2 (Fig. 5). All amino acids selected for site directed mutagenesis (SDM) had hydrophobic side chains, and comprised of 6 valines, 2 phenylalanines, one leucine, one tryptophan, and one tyrosine. Alanine mutations to three amino acid residues, V104, V106 and V110 resulted in a significant reduction in cholesterol binding (p < 0.05) as compared to wild-type Der p 2 (WT Der p 2) (Fig. 6A–C), indicating that these residues are possible cholesterol binding sites (Supplementary Fig. S3). Circular dichroism (CD) spectra of these alanine mutants showed typical β-sheeted secondary structures, similar to wild type (WT) Der p 2 with minor variations of the minima and maxima molar ellipticity intensities (Supplementary Fig. S4). One SDM, V16A demonstrated higher cholesterol binding compared to WT Der p 2, possibly due to localised changes in the formation on the hydrophobic cavity that favours cholesterol binding (Fig. 6D, Supplementary Fig. S3). The remaining seven SDMs showed no significant changes in terms of cholesterol binding (Supplementary Fig. S5).

## Discussion

Lipid binding allergens have been described from diverse sources such as cockroaches (Bla g 1), pollen (Bet v 1), fruits (peach allergen Pru p 3, Brazil nut allergen Ber e 1, peanut allergen Ara h 8) and animals (Cat dander protein Fel d 1, dog allergen Can f 6) among others^15^. In plants, several lipid transfer proteins such as Ole e 7 from olive^16^ and Art v 3 from mugwort weed^17^ are important allergens. Among dust mite allergens, groups 2, 5, 7, 13, 14, 16 and 22 have been reported to be potentially lipid binding^18^. Der p 5 has a large hydrophobic cavity with potential to bind to hydrophobic ligands^19^, while the structure of Der p 7 resembles that of a lipopolysaccharide (LPS)-binding protein^20,21^. Group 13 allergens binds to fatty acids and other lipids such as eisosanoids and retinoids. Group 14 allergens of dust mites are homologous to the large lipid transfer protein (LLTP) family that include apolipophorin or vitellogenin-like proteins, which are proposed to have energy storage and transport functions^22^.

In this study, we show that Der p 2 preferentially binds to cholesterol among the different sterols and lipid molecules tested, and the organic fraction of Der p 2 contained cholesterol, indicating that it is the natural lipid ligand of Der p 2. Some promiscuity in lipid binding was observed as Der p 2 also showed moderate binding to sterols such as lathosterol and 7-ketocholesterol, as well as a phospholipid, palmitoyloleoylphosphatidylcholine (POPC). The promiscuous nature of Der p 2 in binding to its lipid ligand could explain why Der f 2, a closely related allergen with 88% amino acid sequence identity, was shown to bind to LPS in an earlier study^6^. The structural solution of group 2 allergens (Der f 2 and Der p 2) has described both an ‘open’ and ‘closed’ structures, whereby in the ‘open’ structure the two beta sheets are further apart, creating a larger cavity region^8,9,23^. However, even in the ‘open’ conformation which the group 2 allergens naturally adopt, it can only accommodate smaller lipids containing a pair of fatty acids or string like compounds such as polyethelyneglycol (PEG)^8,24^. It was reported that in order to bind to LPS, the cavity of Der f 2 needs to open up much wider by shifting the two beta-sheets further apart and reorienting two aromatic side chains of Tyr-90 and Tyr-92^6^. While group 2 allergens may bind to a range of lipid molecules, our data clearly shows that it shows preferential binding to cholesterol. In addition, two other allergens with structural similarities to Der p 2, namely Der f 2, a well characterized group 2 allergen from *D. farinae* and Der f 22, a paralogue of group 2 allergens^12^ also bind to cholesterol in a dose-responsive manner, similar to Der p 2.

In this study, we show evidence that both native and recombinant Der p 2 bind to cholesterol is provided. The cholesterol bound to native Der p 2 (nDer p 2) could originate from dietary sources of the dust mite *D. pteronyssinus*, which feeds on debris of human skin. In the case of recombinant Der p 2 (rDer p 2) on the other hand, the cholesterol most likely originates from the growth medium used for bacterial cultures used to produce recombinant protein. Indeed, a delipidated fraction of Luria Bertani medium (BioBasic Inc.) used here contained 82 ng/mL of cholesterol, as measured by mass spectrometry (data not shown).

To further investigate the site of cholesterol binding, eleven site-directed mutants of amino acids lining the cavity of Der p 2 were generated. While the secondary structures of these mutants were similar to that of the wild-type Der p 2 as evaluated by the CD spectra, single alanine mutation to three residues, V104, V106 and V110 resulted in a significant difference in cholesterol binding compared to wild-type Der p 2, indicating that these residues were important in the binding of cholesterol to Der p 2. These results were not as drastic as that observed for NPC2^11^, where single alanine mutations of residues F66, Y100 and V96 almost completely abolished binding to cholesterol, perhaps suggesting that lipid recognition in both proteins could be mediated by different mechanisms.

Lipid cargos of other lipid binding allergens have demonstrated the capacity to either promote or inhibit host Th2 responses, thereby affecting allergenicity. For example, two allergens from the lipocalin protein family, Bos d 5 (the major cow milk protein allergen) and Bet v 1 (the major pollen allergen) are able to promote the production of Th2 cytokines in its lipid bound form (apo-form), but not when it is devoid of lipid cargo (holo-form)^25,26^. The presence of exogenous relevant lipids in addition to certain allergens from the cupin superfamily, such as the mustard allergen Sin a 2 and peanut allergen Ara h 1, resulted in the increase of IL-4:IL-10 ratio and IL1β production by human monocyte-derived dendritic cells, thus favouring a pro-inflammatory environment^27^. While effects of the lipid cargo of Der p 2 on its allergenicity are still unknown, a recent study has shown in *in vitro* experiments that Der p 2 can be internalized by human bronchial epithelium, however the role of its lipid ligand in this process remains unclear^28^.

Cholesterol has previously been shown to mediate allergic reactions, which are characterized by increases in serum IgE levels, elevated Th2 and suppressed Th1 cytokines, and increased airway inflammation. Exogenous addition of cholesterol to peripheral blood mononuclear cells of latex allergic patients resulted in increased latex-specific IgE, reduction in Th1 cytokine and increase in Th2 cytokine production^29^. In a murine model of asthma, intraperitoneal injections of cholesterol-lowering agents caused suppression of eosinophilic airway inflammation and reduced the Th2 cytokine levels^30^. These experiments suggest that cholesterol enhances the allergic response in sensitized individuals. In view of the ability of Der p 2 or Der f 2 to bind to several lipids classes and seen from this study and that those reported earlier^6^ it remains to be investigated what are the mechanisms that govern the binding of Der p 2 or other group 2 allergens to their lipid ligand(s), and how does its ligand binding properties affect its potency as an allergen and in its physiological role.

## Methods

### Subcloning and site directed mutagenesis

DNA encoding for Der p 2, Der f 2 and Der f 22 were amplified from cDNA of *Dermatophagoides pteronyssinus* or *D. farinae* using primers containing *BamH* I and *Eco R* I restriction sites. The DNA inserts were ligated into a modified pET-32a plasmid (Novagen) and transformed into DH5-α competent cells. Insert sequences were verified by DNA sequencing. Mutant constructs of Der f 2 were generated using the Quikchange® kit (Stratagene) with primers containing mismatches coding for alanine. Correct substitutions were verified using DNA sequence analysis. Mutated DNA insert was sub-cloned in the same manner as WT Der p 2. Primer sequences used for cloning and site directed mutagenesis are listed in Supplementary Information.

### Expression and purification of wild type allergens and mutant Der p 2

Plasmid containing DNA insert of WT allergens (Der p 2, Der f 2 and Der f 22) or alanine mutants of Der p 2 were transformed into *E. coli* (BL21, DE3) for protein expression. Cultures were induced overnight with 1.0 mM IPTG at 20 °C. The proteins were expressed as a His-tagged soluble protein and purified using a Ni-NTA resin (Novagen) under denaturing conditions. Recombinant proteins were refolded by rapid dilution into 50 mM sodium acetate, pH 4.6 at 4 °C and concentrated using Amicon® Stir Cell (YM3, Millipore).

### Circular dichroism (CD) spectrophotometry

CD spectra was acquired using a J-180 Spectrophotometer (Jasco) using a 1 mm path length quartz cuvette. The spectra were recorded at the resolution of 0.1 nm and averaged for 10 scans (50 nm/min) from 190 to 260 nm.

### Liposome preparation

Ten milligrams of bovine brain extract (Folch fraction 1, Sigma) was dissolved in 5 mL of chloroform: methanol (1:1). This solution was dried to completion under a stream of nitrogen. The dried lipid film was then hydrated in 1 mL rehydration buffer (30 mM Hepes, 100 mM KCl, 0.3 M sucrose), and passed (10 times) through an extruder (Avanti Polar Lipids), with 0.4μm polycarbonate membranes. For preparation of liposomes with exogeneous cholesterol, 2 mg of cholesterol was added to the bovine lipid extract in the first step.

### Detection of liposome binding to Der p 2 by liposome sedimentation and SDS-PAGE

The liposome binding assay was adapted from previously published methodologies^31,32^. Fifty micrograms of Der p 2 or glutathione-S-transferase (GST) of equal volume (30 μL, 1.5 mg/mL) was added to increasing amounts of liposomes (5–40 μL; 10 mg/mL) and incubated for 30 mins at 37 °C. The incubation mixture was then centrifuged at 30,000 rpm for 30 mins at 37 °C. The resulting supernatant and pellet were mixed with equal volume of 2x SDS sample buffer (200 mM Tris-Cl (pH 6.8), 400 mM DTT, 8% SDS, 0.4% Bromophenol blue, 50% glycerol) and boiled for 10 mins. The samples were then separated on a 12% SDS-PAGE gel followed by Coomassie Blue staining. GST was used as a negative control in this experiment.

### Lipid ELISA

The cholesterol binding assay was modified from Kobayashi *et al*. (2001). Microtiter plate wells (Maxisorp, Nunc) were coated with lipids (0.02–6.40 nmol/well) by ethanol evaporation at room temperature. The wells were blocked with PBS containing 1% BSA for 1 hr prior to incubation with 0.5 μg/mL of protein (100 μL/well) for 1 hr. Binding of protein was then detected using specific polyclonal anti-rabbit IgG-antibodies produced in house by immunizing New Zealand White rabbits by subcutaneous injection with 300 μg of the recombinant protein with Freund’s adjuvant. Alkaline phosphatase labelled anti-IgG antibodies were then added, the colour was developed using PnPP (p-Nitrophenyl Phosphate) substrate (Sigma), and absorbance was measured at 405 nm. The microtiter plate was washed three times between each step using PBS. Native Der p 2 used was obtained from Indoor Biotechnologies (USA). The intra-assay variations were less than 5% for all assays and the inter-assay variations ranged from 6.4% to 13.7% (depending on the allergen-lipid combination).

### Ethical approval for animal immunizations

The animal immunization protocols were reviewed and approved by the Institutional Review Board of the National Healthcare Group Domain Specific Review Board - B/04/055 and the Animal Research Ethics Committee of NUS, and are in compliance with the Helsinki declaration.

### Extraction of lipid fraction from Der p 2

Three hundred microliters of chloroform: methanol (1:2) was added to 100 μL of Der p 2 (1.5 mg/mL), followed by the addition of 400 μL of chloroform and 300 μL of 1 M KCl. The mixture was vortexed hard for 1–2 mins and centrifuged for 30 s at 13200 rpm. The chloroform layer (bottom) was recovered and transferred to a fresh tube. The organic solvent was dried. Samples were next resuspended in chloroform: methanol (1:1) for mass spectrometry.

### HPLC/APCI/MS/MS analysis of cholesterol

Analysis of cholesterol was carried out using atmosphere pressure chemical ionization (APCI) with an Applied Biosystems 4000 Q-Trap mass spectrometer operated in the positive mode (Applied Biosystems). The APCI conditions were set at 500 °C (vaporizer temperature) and 3 μA (corona discharge current). Samples were introduced into mass spectrometer after separation on an Agilent Zorbax Eclipse XDB-C18 column (i.d. 4.6 × 150 mm) using an Agilent 1100 liquid chromatography (LC) system (Agilent Technologies). The chromatographic conditions are (1) chloroform/methanol (1:1) as a mobile phase at a flow rate of 500 μL. min-1; (2) column temperature: 25 °C; (3) injection volume: 20 μL. Tandem mass spectra of ions at m/z 369.3 for both cholesterol standards and samples, and ions at m/z 375.1 for d6-cholesterol standard were acquired with a collision energy of 40 ev. Multiple reaction monitoring, MRM, of parent/fragment ions were used for quantification of cholesterol (369/161) and deuterated cholesterol standard (375/161), respectively. Analysis of polar lipids was carried out using electrospray ionization (ESI) instead of APCI. Samples were directly introduced into the mass spectrometer using chloroform/methanol (1:1) as a mobile phase at a flow rate of 200 μL/min. Twenty microliters of sample or standard mixture was injected to obtain lipid profiles and to estimate contents of potential lipids in the extract.

### Statistical analysis

Statistical analysis was carried out using GraphPad Prism version 6.0. Comparisons between Der p 2 binding to different lipids and binding of cholesterol to different site-directed mutants were performed using paired *t*-test (p < 0.05, two tailed).

## Supplementary information

Supplementary Information
