# Supplementary material for: The major allergen Der p 2 is a cholesterol binding protein
Source: Sci Rep. 2019 Feb 7;9:1556. doi: 10.1038/s41598-018-38313-9 (PMC6367342; doi:10.1038/s41598-018-38313-9)
Supplement: Supplementary file 1 — Supplementary Information [file 41598_2018_38313_MOESM1_ESM.pdf]

The major allergen Der p 2 is a cholesterol binding protein

Short title: Der p 2 is a cholesterol binding protein

Kavita Reginald[1] and Chew Fook Tim[2]*

[1]Department of Biological Sciences, Sunway University, Bandar Sunway 47500, Selangor, Malaysia

[2]Allergy and Molecular Immunology Laboratory, Department of Biological Science, National University of Singapore, 117543 Singapore

Corresponding author:

Dr. Chew Fook Tim, Allergy and Molecular Immunology Laboratory, Lee Hiok Kwee Functional Genomics Laboratories, Department of Biological Sciences, 14 Science Drive 4, National University of Singapore, 117543 Singapore, Tel. +65-65161685; Fax +65-67792486; E-mail: dbscft@nus.edu.sg

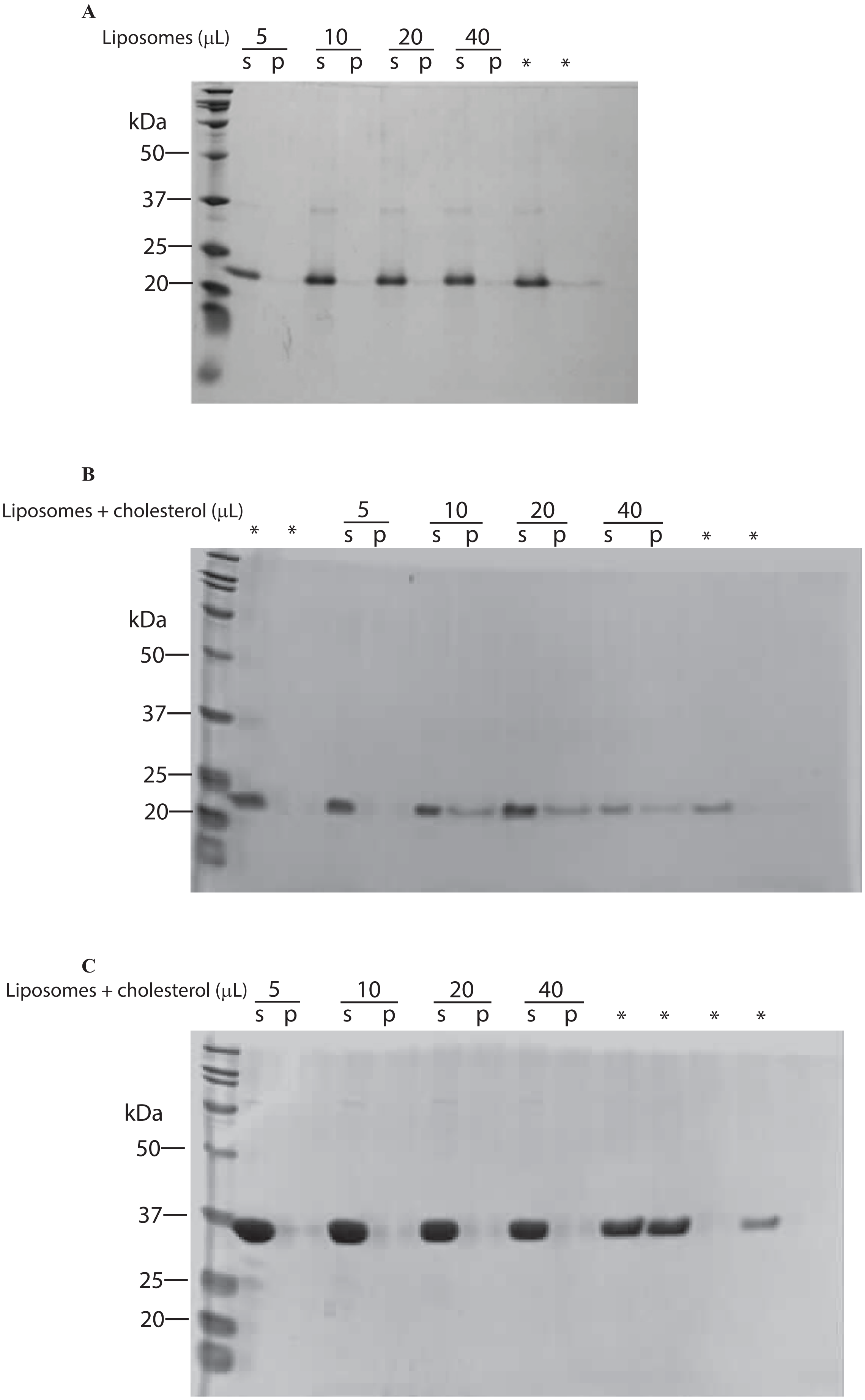

Supplementary Figure S1. Liposome pull down assay. Liposomes with a fixed diameter of 0.4μm were prepared using bovine brain lipid extract in HEPES-KCl buffer with 0.3M sucrose, with or without exogenous cholesterol 20% (w/w). Increasing amounts of liposomes (5, 10, 20 or 40 μL of 2 mg/mL lipid suspension) were incubated with 50μg of Der p 2 (A, B), or gluthation-S-transferase (C) for 30 minutes at 37°C. The incubation mixture was then centrifuged at 30,000 rpm and the resulting supernatant (s) and pellet (p) were seperated on a 12% SDS-PAGE gel, and stained with Coomasie Blue. Full length gels are depicted. Astericks (*) represents samples that were not included in this study.

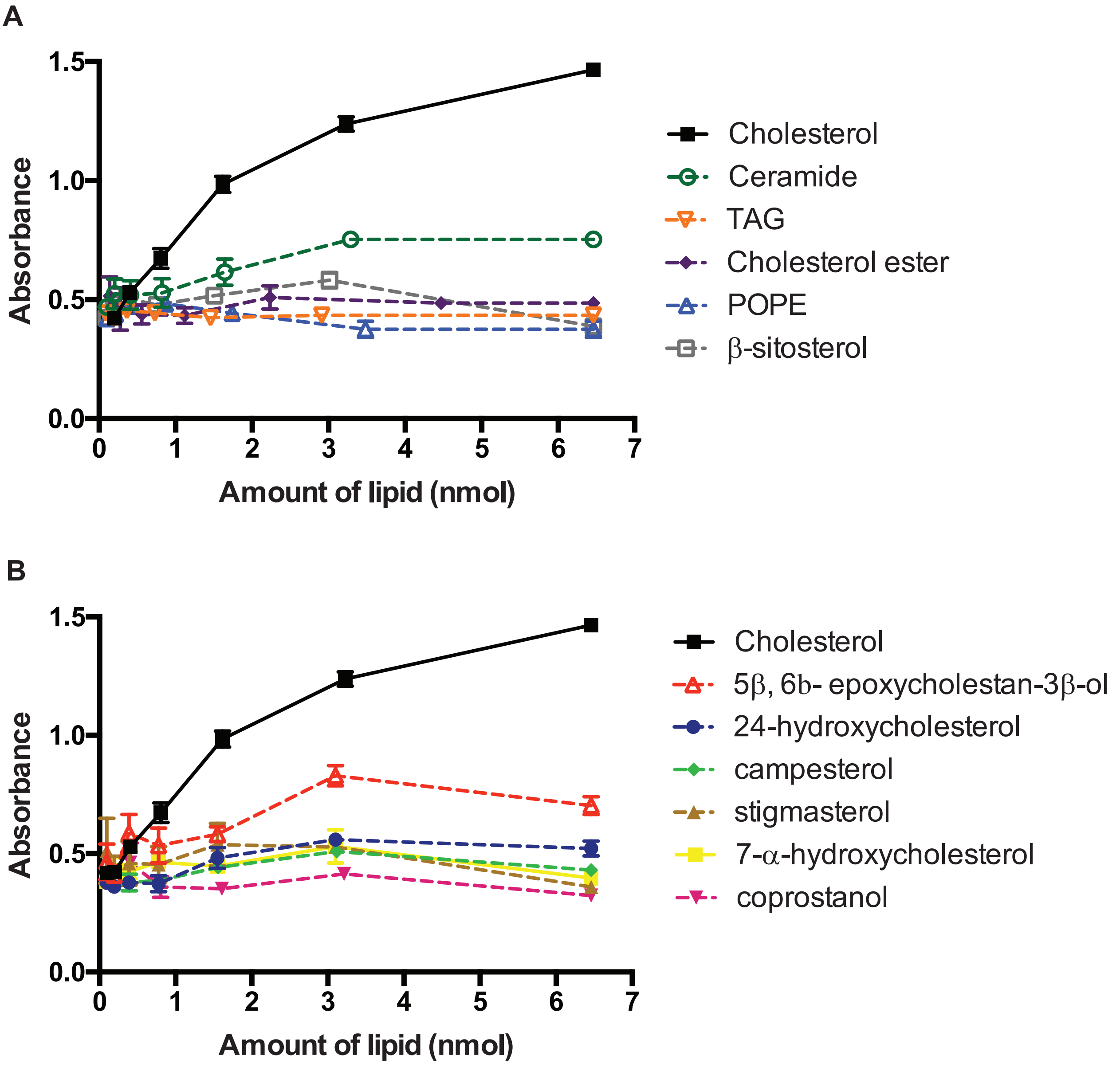

Supplementary Figure S2. Binding of recombinant Der p 2 to sterols, phospholipids and sphingolipids. Lipids were coated (in serial dilutions) onto microtiter plates and incubated with 0.5ug/mL of Der p 2. The bound Der p 2 was then probed with anti Der p 2 IgG antibodies, followed by anti-IgG linked to alkaline phosphatase. Absorbance was measured at 405nm after addingthe PnPP substrate. Standard errors of duplicate experiments are shown. POPE, palmitoyloleoylphosphatidylglycerol; TAG, triacylglycerol (1,2-dioleoyl-3-palmitoyl-sn-glycerol), ceramide ((2S,3R,4E)-2-acylamino-1,3-octadec-4-enediol).

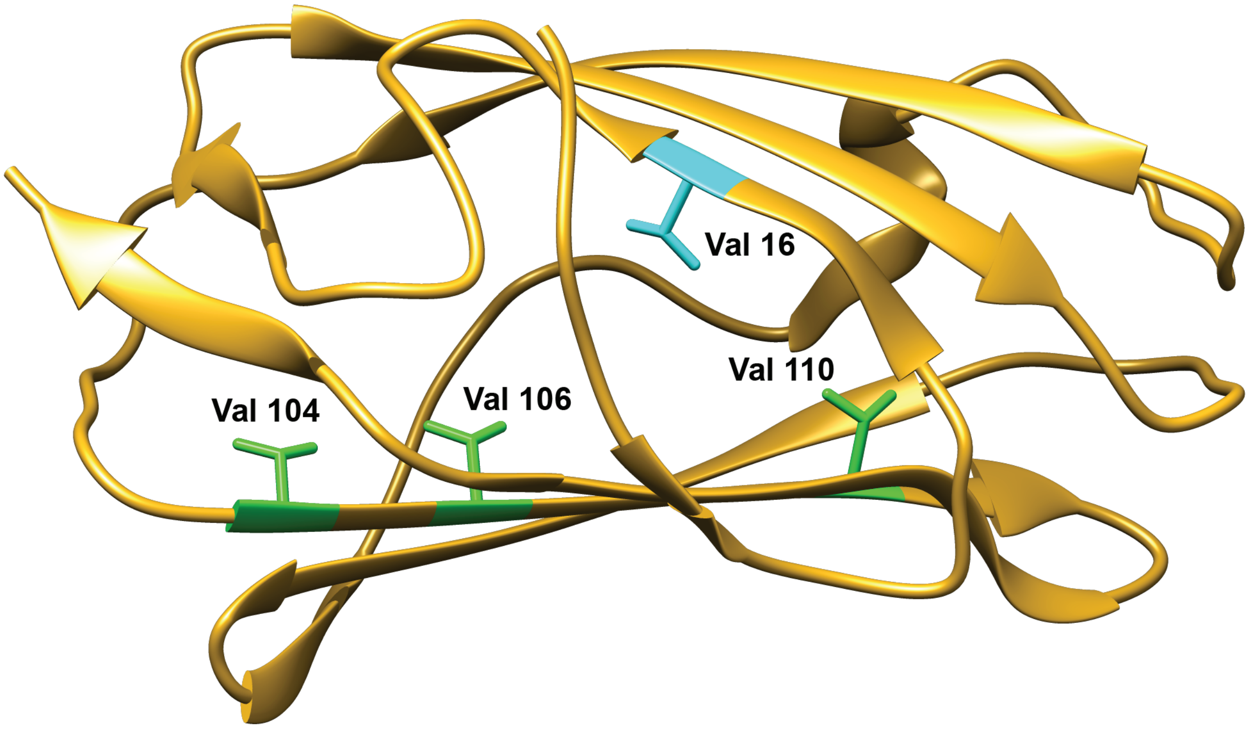

Supplementary Figure S3. Ribbon diagram of the solved structure of Der p 2 (PDB: 1KTJ, chain A). Mutations to V104, V106 and V 110 (shown in green) to alanine resulted in significant reduction in cholesterol binding compared to wild-type Der p 2, whereas mutation of V16 to alanine (cyan) caused an increase in cholesterol binding.

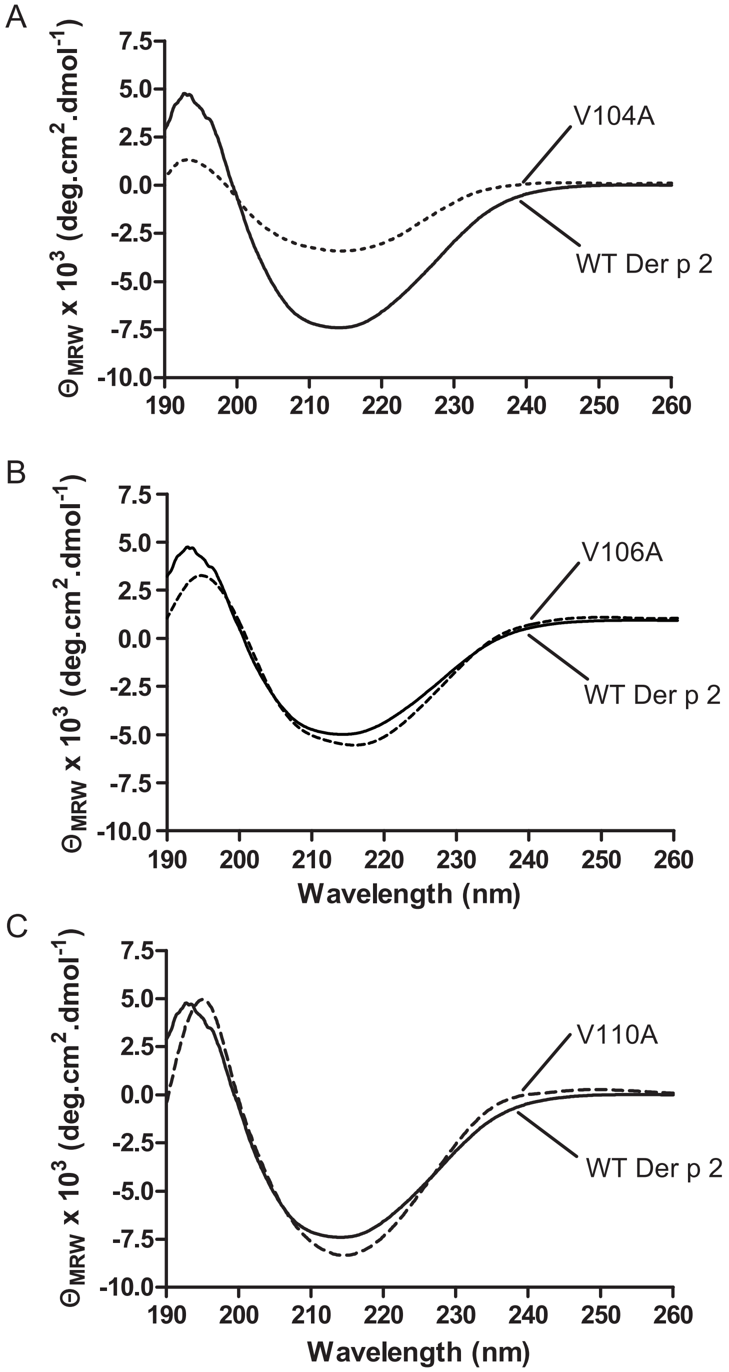

Supplementary Figure S4. Circular dichroism spectra of wild type (WT) Der p 2 and (A) mutant V104A, (B) mutant V106A and (C) mutant V110A. Spectra were recorded using 20 μM solutions of each protein in 50 mM sodium acetate pH 4.6 at room temperature. Average spectra from 10 scans are shown.

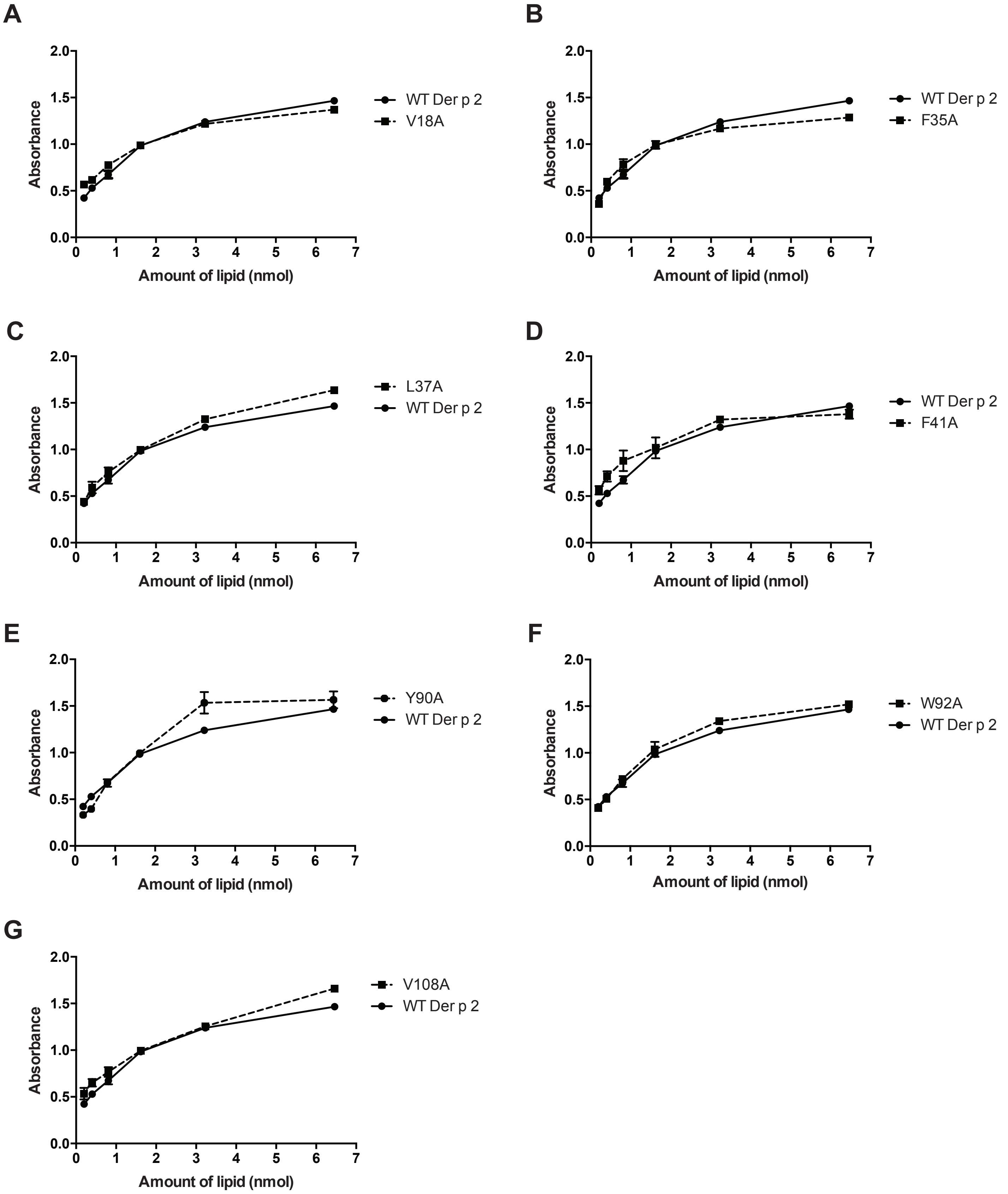

Supplementary Figure S5. Binding of single site directed mutants of Der p 2 to cholesterol. The binding of the seven different alanine mutants (A-G) in relation to wild type (WT) Der p 2 to cholesterol was performed using an ELISA based assay. Mean and standard errors of duplicate experiments are shown.
